# An Audit of the Physical Health Assessment of Patients Following Admission to the General Adult Inpatient Wards in Mersey Care NHS Foundation Trust

**DOI:** 10.1192/bjo.2026.11698

**Published:** 2026-06-30

**Authors:** Natasha Hall, Beth Jones, Seerat Shirazi, Rachel Rosewicz, Declan Hyland

**Affiliations:** Mersey Care NHS Foundation Trust, Liverpool, United Kingdom

## Abstract

**Aims:**

Individuals with severe and enduring mental illness (SMI) are at greater risk of poor physical health than the general population and have a higher premature mortality rate. Average life expectancy of individuals with SMI is ~15 - 20 years shorter than the general population, often due to physical disease, most commonly of metabolic or cardiovascular origin. A significant contributor is underperformance of physical examination, assessment and intervention.NICE Clinical Guidelines and Mersey Care NHS Foundation Trust guidelines state that, following admission to a mental health inpatient unit, a patient should have a physical examination and assessment completed within 24 hours.

This audit assessed if the following were completed within 24 hours of admission to the Trust’s general adult inpatient wards: physical examination; set of physical observations; Body Mass Index (BMI); Malnutrition Universal Screening Tool (MUST); smoking status; alcohol consumption status; and physical health comorbidity (cardiac disease, respiratory disease, diabetes mellitus) status documented.

**Methods:**

A retrospective audit was conducted, with the sample comprising all patients on 12 of the Trust’s general adult inpatient wards on a specific date in December 2025.An audit tool was designed with standards based on NICE Clinical Guidelines and the Trust’s physical health policy and completed for each patient using the patient’s electronic patient record.

**Results:**

129 of the 200 patients were male, 71 were female (3 patients were born biologically male but transitioned to female).Age of patients ranged from 18 to 73 years.Within 24 hours of admission to the ward, 60% of patients had a physical examination completed; 86% had a set of physical observations completed; 62% had a BMI done; 62% had a MUST score done; 54% had a smoking status recorded; 65% had their alcohol consumption recorded; and 67% of patients had their physical health comorbidity status checked.

**Conclusion:**

Improvement is required to ensure patients have a complete physical examination done within 24 hours of admission, and, or those declining one, this should be documented and re-attempted at the earliest opportunity. Timely completion of a physical examination ensures rapid identification of any patient that is acutely physically unwell at the time of admission. Ensuring the alcohol consumption status is checked will reduce risk of acute alcohol withdrawal and smoking status should be checked to enable prompt consideration of nicotine replacement therapy. There is a need for greater awareness from both medical and nursing staff on the ward of the importance of physical health assessment.

